# Multimorbidity Patterns and Mortality Risk in a National Sample of US Adults Identified Using Latent Class Analysis

**DOI:** 10.3390/diseases14080270

**Published:** 2026-07-24

**Authors:** Emmanuel U. Azu, Gulzar H. Shah, Toktam Naderimoghaddam, Lili Yu

**Affiliations:** 1 Department of Biostatistics, Epidemiology and Environmental Science, Jiann-Ping Hsu College of Public Health, Georgia Southern University, Statesboro, GA 30458, USA; ea07407@georgiasouthern.edu (E.U.A.); tn01534@georgiasouthern.edu (T.N.); lyu@georgiasouthern.edu (L.Y.); 2 Department of Health Policy & Community Health, Jiann-Ping Hsu College of Public Health, Georgia Southern University, Statesboro, GA 30458, USA

**Keywords:** latent class analysis, multimorbidity, mortality, National Health Interview Survey, National Death Index, United States adults

## Abstract

Background/Objectives: Multimorbidity is increasingly recognized as a major contributor to mortality worldwide, yet its underlying patterns and prognostic implications remain poorly understood in the United States. This study identified distinct multimorbidity patterns and examined their association with all-cause mortality in a nationally representative sample of U.S. adults. Methods: We conducted a retrospective cohort study using data from the 2004 National Health Interview Survey linked to the National Death Index through 2019 (n = 28,598). Latent class analysis identified unobserved multimorbidity classes based on patterns of co-occurring physician-diagnosed chronic conditions, and Cox proportional hazards models were fitted to estimate mortality risk while accounting for complex survey design. Six distinct multimorbidity classes were identified, reflecting cardiometabolic, respiratory, cardiovascular, and inflammatory disease profiles. Results: Compared with the Low Multimorbidity group, all other classes were associated with increased mortality risk. In fully adjusted analyses, the Severe Cardiopulmonary–Metabolic (HR 3.71, 95% CI 2.99–4.60) and Advanced Cardiovascular (HR 2.81, 95% CI 2.52–3.14) classes showed the highest risks. Intermediate risks were observed in the Cardiometabolic–Arthritis (HR 2.45, 95% CI 2.13–2.81) and Respiratory–Musculoskeletal (HR 1.39, 95% CI 1.22–1.58) classes, while the Inflammatory Pain–Airway class showed a more modest increase. Subgroup analyses suggested stronger relative effects among younger adults and women in the most severe classes. Conclusions: The study findings highlight the heterogeneous nature of multimorbidity and suggest that specific disease clusters carry substantially different mortality risks. Recognizing these patterns may improve risk stratification and support more targeted, patient-centered care strategies.

## 1. Introduction

Chronic non-communicable diseases (NCDs) have been identified as the leading cause of death worldwide. The World Health Organization has identified heart disease, cancer, respiratory issues, and diabetes as contributors to roughly 75% of all deaths worldwide [1]. A growing proportion of this burden is not due to single conditions but to the coexistence of multiple chronic diseases within individuals, a phenomenon known as multimorbidity [2,3]. Multimorbidity is seen as the presence of two or more chronic conditions, although this definition varies across studies [4]. Regardless of definition, its prevalence is steadily rising due to population aging, improved survival from acute events, and the widespread presence of lifestyle-related risk factors [5]. Recent systematic reviews and meta-analyses estimate that, due to an aging population, between one-third and nearly half of adults in community settings worldwide live with multimorbidity [6,7], with higher prevalence among older adults, women, and those in socioeconomically disadvantaged groups [8].

The implications of multimorbidity extend beyond disease counts. Nunes et al. reported that individuals with multimorbidity experience reduced quality of life, functional decline, greater psychological distress, and substantially higher healthcare utilization and costs [9,10]. What is becoming clearer over time is just how closely it ties to mortality. Several longitudinal studies have shown that people living with multiple chronic conditions tend to face a much higher risk of dying earlier than expected, even after accounting for factors such as age, demographics, and lifestyle [8,9,10,11,12]. These associations highlight multimorbidity as not only a clinical management challenge but also a key determinant of population survival.

Despite its growing importance, much of the existing literature on multimorbidity adopts a reductionist approach, focusing on individual diseases or broad condition counts [13,14,15]. While such measures are straightforward to compute, they do not adequately capture the underlying clustering of chronic conditions within individuals. Chronic diseases often co-occur in systematic, non-random patterns driven by shared biological mechanisms, lifestyle factors, and social determinants, and these groupings may have synergistic effects on health outcomes [16]. For instance, clusters characterized by cardiometabolic conditions, such as diabetes, obesity, and cardiovascular disease, may carry different prognostic implications compared to those dominated by mental health or musculoskeletal disorders [17,18]. Recognizing these patterns is essential for improving risk stratification, informing targeted interventions, and supporting more effective health system planning.

Latent class analysis (LCA) offers a robust, data-driven method for identifying unobserved subgroups within populations based on shared patterns of conditions [19,20]. Unlike simple counts, LCA accounts for the heterogeneity of multimorbidity by uncovering distinct classes of individuals who share similar disease profiles. This approach enables researchers to move beyond the “number of conditions” paradigm and instead characterize clinically meaningful phenotypes of multimorbidity [21]. Recent applications of LCA in health research have revealed that multimorbidity is far from homogeneous. For instance, studies in Europe, China, and Latin America have identified clusters such as cardi-ometabolic, respiratory, and psychiatric profiles, each with differential impacts on survival [22,23,24]. Importantly, these findings suggest that some multimorbidity classes may carry disproportionately high risks of mortality, while others are less prognostically severe, underscoring the need for nuanced approaches to patient management [21].

Nonetheless, important gaps remain. Much of the available evidence has been generated outside the United States or within narrowly defined clinical populations, such as individuals with osteoarthritis or ischemic heart disease [17,21,25,26]. While these studies have provided valuable insights, their findings may not readily generalize to the broader U.S. adult population, where differences in healthcare systems, population structure, and social determinants of health shape patterns of disease. In contrast, national surveillance data indicate that multimorbidity is highly prevalent in the United States, affecting more than 40% of adults and over two-thirds of those aged 65 years and older [27,28]. This burden is not evenly distributed; prior work has shown that socioeconomically disadvantaged populations and racial and ethnic minorities experience disproportionately higher rates of multimorbidity, reflecting persistent inequities in health outcomes [29]. Despite the magnitude of this public health challenge, relatively few studies have leveraged nationally representative U.S. data to examine how distinct patterns of multimorbidity relate to mortality risk.

Addressing these gaps is therefore both timely and necessary. Identifying clinically meaningful multimorbidity classes within U.S. adults and quantifying their associations with survival outcomes can offer important insights for both clinical practice and health policy. From a clinical standpoint, understanding which clusters of conditions confer the greatest mortality risk may help guide preventive strategies, improve disease management, and support more informed patient counseling. From a policy perspective, such evidence can inform the allocation of healthcare resources and the development of targeted interventions for high-risk populations. Moreover, analyses based on nationally representative data enhance the generalizability of findings, ensuring that conclusions are applicable to the diverse U.S. population and, importantly, relevant to ongoing efforts to reduce health disparities.

Therefore, the present study aims to identify latent classes of multimorbidity in a large, nationally representative sample of U.S. adults, and evaluate the association between these multimorbidity classes and all-cause mortality. By doing so, this research contributes to the growing body of literature on multimorbidity and survival, while filling a critical gap in population-based evidence from the United States.

## 2. Materials and Methods

### 2.1. Study Design and Data Source

We conducted a retrospective cohort study using the latest publicly available pooled data from the U.S. National Health Interview Survey (NHIS) 2004, linked to the National Death Index (NDI), with follow-up through 31 December 2019. The NHIS is an annual, nationally representative survey of the non-institutionalized U.S. civilian population conducted by the National Center for Health Statistics (NCHS). It employs a multistage, stratified-cluster probability design to capture health behaviors, chronic conditions, and sociodemographic characteristics.

Mortality linkage was performed by the National Center for Health Statistics (NCHS) using a validated probabilistic record linkage algorithm that matches NHIS participants to National Death Index (NDI) records based on multiple personal identifiers. Only respondents eligible for mortality linkage were included in the linked mortality file. Detailed descriptions of survey methodology and linkage procedures are publicly available from NCHS (ftp://ftp.cdc.gov/pub/Health_Statistics/NCHS/datalinkage/linked_mortality/archived_files/mort_2004_release accessed on 26 February 2026).

### 2.2. Study Population

**Figure 1:**
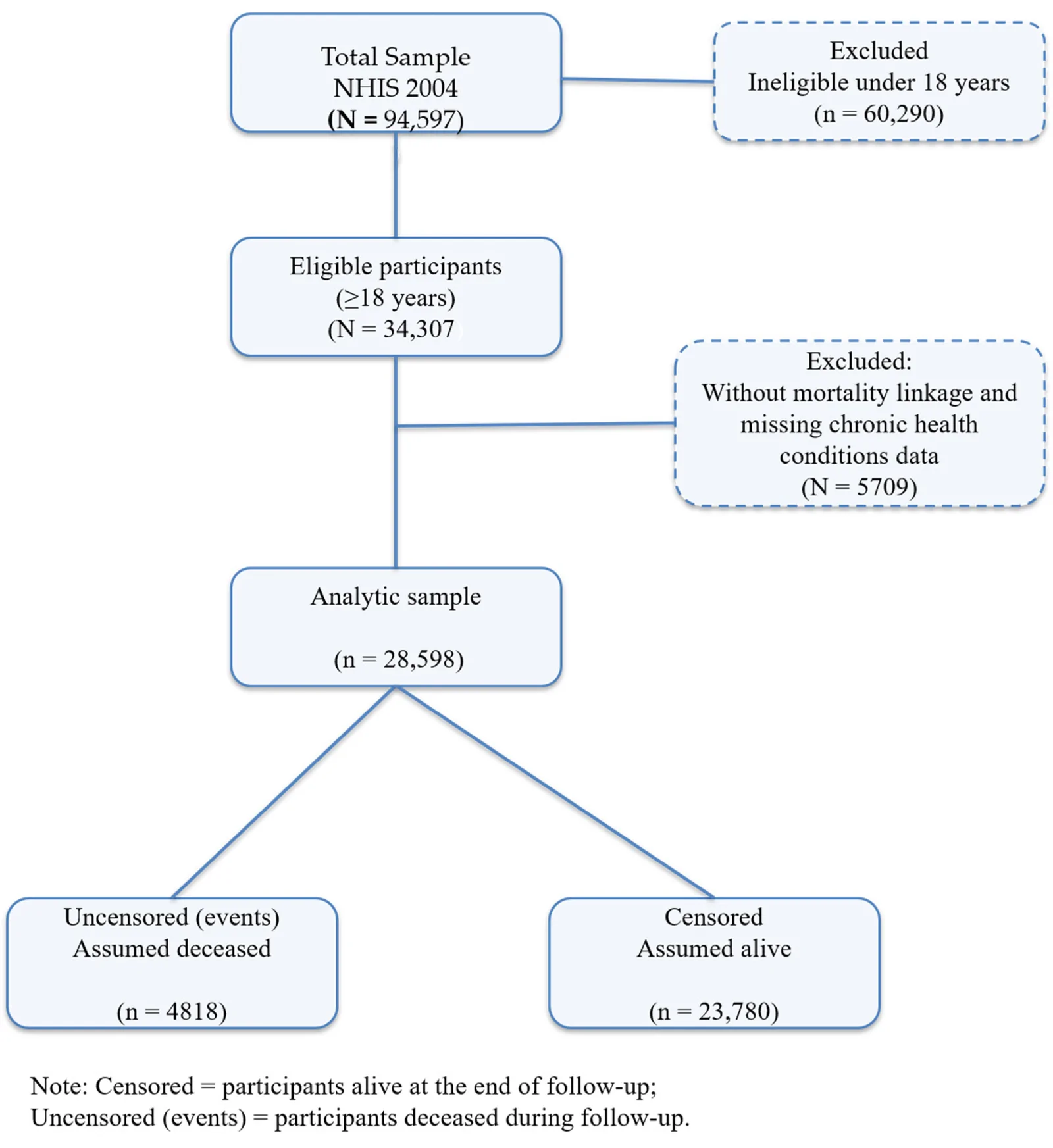
Flowchart of eligible and ineligible participants.

This study analyzed data from the 2004 National Health Interview Survey (NHIS), which included 94,597 participants. Individuals who were younger than 18 years or otherwise ineligible for mortality linkage were excluded, leaving 28,598 eligible adults with linked mortality data and complete information on the chronic health conditions included in the latent class analysis. The participant selection process is presented in Figure 1. Participants were followed from the date of their NHIS interview until death or administrative censoring on 31 December 2019. During follow-up, 4818 participants died and 23,780 remained alive at the end of follow-up and were administratively censored. To assess the representativeness of the final analytic sample following exclusions, the weighted baseline characteristics of the full eligible sample and the final analytic sample were compared (Supplementary Table S1).

### 2.3. Mortality Outcome

The primary outcome was all-cause mortality. Vital status and date of death were ascertained through NHIS–NDI linkage. Survival time was defined as the number of months from the NHIS interview date to death or censoring. Participants alive at the end of follow-up were administratively censored.

### 2.4. Chronic Conditions

Chronic conditions were assessed based on respondents’ self-reported history of physician diagnosis. Participants were asked whether a doctor or other health professional had ever informed them that they had specific chronic conditions. Following prior multimorbidity research and the availability of variables in NHIS, we included hypertension, diabetes, coronary heart disease, stroke, cancer, chronic obstructive pulmonary disease, asthma, chronic kidney disease, liver disease, depression, and hepatitis, as well as arthritis, which in the NHIS encompasses arthritis, rheumatoid arthritis, gout, lupus, and fibromyalgia. All conditions were coded as binary indicators (present/absent) based on self-reported lifetime physician diagnosis. Conditions with very low prevalence (<2%) were excluded to improve model stability.

Multimorbidity was operationalized not by condition count but through latent class analysis (see Table 1 below), allowing identification of disease clusters.

### 2.5. Covariates

We adjusted all analyses for sociodemographic and behavioral factors known to confound the association between multimorbidity and mortality. Sociodemographic covariates included age (continuous), sex (male/female), race/ethnicity (non-Hispanic White, non-Hispanic Black, Hispanic, or other), marital status (married, widowed/divorced/separated, or never married), educational attainment (<high school, high school graduate, >high school), and family income-to-poverty ratio defined as the ratio of total family income to the U.S. Census Bureau poverty threshold for the corresponding family size and survey year, with values below 1.0 indicating income below the federal poverty level. Lifestyle covariates comprised smoking status classified according to NHIS definitions as never, former, or current smoker, alcohol consumption categorized according to NHIS definitions as none, moderate, or heavy drinking based on respondents’ reported alcohol use frequency and quantity, and body mass index (BMI, kg/m^2^), categorized as underweight, normal, overweight, or obese. These variables were selected based on their availability in the NHIS dataset and prior evidence linking them with both multimorbidity and survival outcomes.

### 2.6. Statistical Analysis

The baseline characteristics of participants were summarized using weighted means and standard deviations for continuous variables and weighted percentages with 95% confidence intervals for categorical variables, reported overall, by mortality status, and by multimorbidity classes.

Latent class analysis (LCA) was used to identify distinct patterns of co-occurring chronic diseases. The method classifies individuals into mutually exclusive latent classes based on responses to a set of binary indicators; in this study, each chronic condition was coded as present or absent. A key assumption of LCA is conditional independence, meaning that within each latent class, the observed indicators are assumed to be independent of one another given class membership. Models were fitted sequentially, beginning with a two-class solution and increasing the number of classes. To reduce the risk of convergence to local maxima, models were estimated using multiple random starting values, and the solution with the highest log-likelihood was retained. At each step, model fit was evaluated using the Akaike Information Criterion (AIC), the Bayesian Information Criterion (BIC), the sample-size-adjusted BIC (SABIC), and entropy, which reflects the clarity of class separation. All four indices were considered jointly in selecting the final model. These indices were not used as a single decision rule; instead, they were compared collectively to identify the smallest model that demonstrated both good statistical fit and adequate class separation. When the indices pointed to different solutions, we prioritized models with lower BIC and higher entropy, consistent with recommendations for latent class model selection [30,31,32,33].

After determining the optimal number of classes, participants were assigned to the class with the highest posterior membership probability. Class characteristics were summarized using item-response probabilities, and classes were labeled according to the conditions most strongly represented within each class. To assess classification quality, several standard diagnostic measures were examined, including the average posterior probabilities (AvePP) for each class, overall entropy, the odds of correct classification (OCC), and the estimated misclassification rate. Together, these metrics provide insight into class stability, separation, and assignment accuracy. Consistent with prior studies [32,33], item-response probabilities below 0.4 were interpreted as indicating low relevance of a condition within a class, values between approximately 0.4 and 0.7 as moderate, and values exceeding 0.7 as strongly characteristic. The item-response probabilities defining the clinical profiles of each class are presented in Table 1.

To evaluate the association between multimorbidity patterns and all-cause mortality, Cox proportional hazards regression models were fitted using survey-weighted follow-up time. Hazard ratios (HRs) and 95 percent confidence intervals (CIs) were estimated with the healthiest class serving as the reference group. Models were built sequentially: first unadjusted, then adjusted for sociodemographic variables, and finally adding behavioral and lifestyle factors. Schoenfeld residuals were examined to assess the proportional hazards assumption, and no meaningful violations were detected. All analyses incorporated NHIS survey weights, strata, and primary sampling units to ensure national representativeness. Latent class analysis (LCA) was conducted without applying survey weights due to software limitations of poLCA; however, all subsequent regression and survival analyses accounted for the complex survey design. LCA was performed in R (v4.4.1; R Foundation for Statistical Computing, Vienna, Austria) using the poLCA package (v1.6.0.1), and survival analyses were conducted using the survival package (v3.8-3). Statistical significance was defined as a two-sided *p* value less than 0.05.

The study was reviewed by the Georgia Southern University Institutional Review Board (IRB) and determined to be exempt from full IRB review under Exemption Category 4 for secondary research involving identifiable private information that was recorded in a manner that did not permit identification of individual participants. This is so because the study used de-identified secondary data from the National Health Interview Survey (NHIS) linked to the National Death Index (NDI). The study was approved under Protocol H26369 on 12 June 2026. All procedures were conducted in accordance with applicable ethical standards and institutional requirements.

The study did not involve the use of GenAI. It was not used to generate scientific content but Grammarly Pro was used solely to assist with language editing and manuscript preparation, including grammar, spelling, punctuation, and readability improvements. All scientific content, analyses, interpretations, and conclusions were developed and reviewed by the authors.

## 3. Results

### 3.1. Prevalence of Chronic Conditions in the Population by Baseline Characteristics and Latent Class

Each participant was assigned to a latent class based on the best-fitting model. The six classes were named according to the dominant patterns of chronic conditions observed within each group: Class 1 (Cardiometabolic–Arthritis), Class 2 (Respiratory–Musculoskeletal), Class 3 (Advanced Cardiovascular), Class 4 (Severe Cardiopulmonary–Metabolic), Class 5 (Inflammatory Pain–Airway), and Class 6 (Low Multimorbidity reference group).

**Figure 2:**
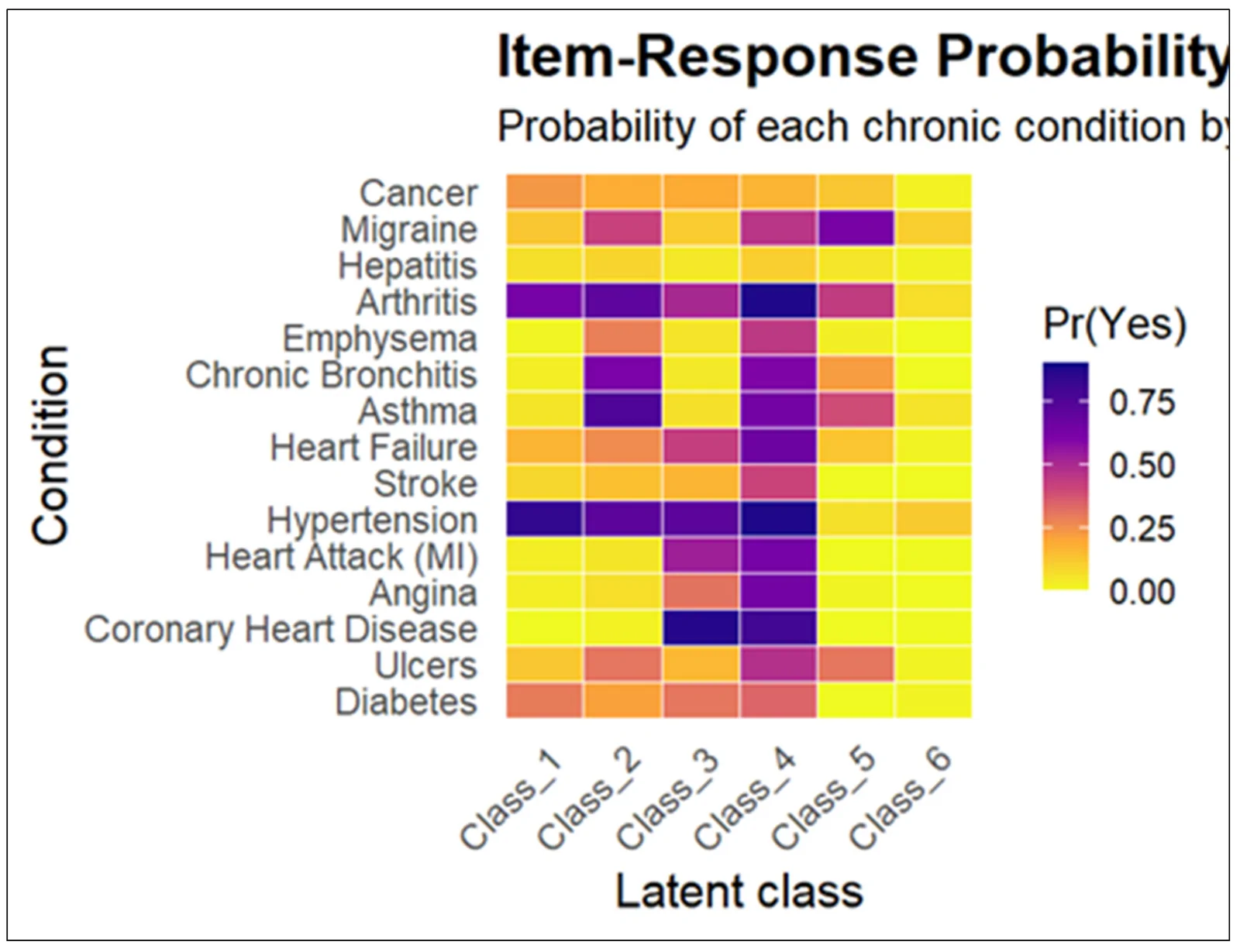
Class Probability Heatmap.

Figure 2 provides a visual overview of the latent disease profiles, whereas Table 1 presents the corresponding item-response probabilities used to define each latent class, while Table 2 describes the corresponding sociodemographic and lifestyle characteristics.

Of the total sample (n = 28,598), most participants were classified into Class 6 (n = 19,695; 68.9%), representing the Low Multimorbidity group. This class consistently exhibited the lowest prevalence across all chronic conditions (Table 1). Participants in this group were also younger (mean age 41.25 years [15.08]), had a balanced sex distribution, and showed more favorable health profiles, including higher proportions with normal BMI (44.0%) and adequate sleep duration (7–8 h: 65.7%) (Table 2).

Class 1 (15.1% of the sample) was characterized by a predominance of cardiometabolic and musculoskeletal conditions and was labeled the Cardiometabolic–Arthritis class. This group showed a high prevalence of hypertension (83.1%), arthritis (63.0%), diabetes (29.3%), and cancer (23.1%), alongside moderate levels of cardiovascular complications such as heart failure (16.6%) and stroke (8.6%) (Table 1). Individuals in this class were older (mean age 61.26 years [14.02]), predominantly female, and more likely to be obese and physically inactive (Table 2).

Class 2 (3.1%) represented a distinct Respiratory–Musculoskeletal profile. It was marked by a high burden of respiratory diseases, including asthma (75.7%), chronic bronchitis (60.8%), and emphysema (28.4%), in addition to substantial arthritis (71.1%) and migraine (41.5%) prevalence (Table 1). Although hypertension was also common, the defining feature of this class was the clustering of airway and musculoskeletal conditions. Participants in this class were generally middle-aged (mean 57.38 years [15.16]), predominantly female, and more likely to report obesity and shorter sleep duration (Table 2).

Class 3 (4.4%), labeled Advanced Cardiovascular, had the highest burden of severe cardiovascular disease. This included a very high prevalence of coronary heart disease (86.4%), myocardial infarction (53.3%), angina (31.2%), heart failure (42.4%), and stroke (16.6%), along with elevated hypertension and diabetes (Table 1). This group had the oldest participants (mean age 66.08 years [12.22]) and the highest proportion of men. It also showed the greatest proportion of former smokers, reflecting cumulative exposure to cardiovascular risk factors (Table 2).

Class 4 (0.7%), the Severe Cardiopulmonary–Metabolic group, represented the most clinically complex phenotype. This class showed widespread, high prevalence across nearly all chronic conditions, spanning the cardiovascular, respiratory, and metabolic domains. Many conditions exceeded 50% prevalence, with hypertension and arthritis approaching 90% (Table 1). Participants in this group were older (mean age 64.03 years [11.99]), predominantly female, and exhibited high levels of inactivity, prior alcohol use, and social vulnerability, including higher proportions of individuals who were separated, divorced, or widowed (Table 2).

Class 5 (7.9%), labeled Inflammatory Pain–Airway, was defined by a different pattern, with a high prevalence of migraine (62.6%) and moderate levels of airway disease (asthma 39.0%, bronchitis 22.1%), alongside intermediate arthritis prevalence (43.6%) and relatively low cardiometabolic burden (Table 1). Participants in this class were younger (mean age 43.64 years [15.26]), predominantly female, and more likely to have normal BMI and to abstain from alcohol (Table 2).

### 3.2. Association Between Latent Classes and All-Cause Mortality

Table 3 presents the results of the Cox proportional hazards models examining the association between latent classes and all-cause mortality. Over the follow-up period from January 2004 to December 2019, a total of 4818 deaths were observed. In the unadjusted analysis, all multimorbidity classes were associated with significantly higher mortality risk compared with the Low Multimorbidity group. The greatest risks were observed in Class 4 (Severe Cardiopulmonary–Metabolic; HR 12.3, 95% CI 10.2–14.7) and Class 3 (Advanced Cardiovascular; HR 9.71, 95% CI 8.90–10.6). Elevated risks were also observed for Class 1 (HR 5.95, 95% CI 5.31–6.68) and Class 2 (HR 5.26, 95% CI 4.91–5.62), while Class 5 showed a smaller but still significant increase (HR 1.39, 95% CI 1.22–1.58).

Adjustment for sociodemographic characteristics attenuated these associations but did not eliminate them (Model 2). Compared with the reference group, Class 4 remained associated with the highest mortality risk (HR 4.10, 95% CI 3.41–4.94), followed by Class 3 (HR 2.99, 95% CI 2.72–3.27), Class 1 (HR 2.70, 95% CI 2.41–3.04), and Class 2 (HR 1.97, 95% CI 1.83–2.11). The Inflammatory Pain–Airway class also remained significantly associated with increased mortality risk (HR 1.29, 95% CI 1.13–1.46).

**Figure 3:**
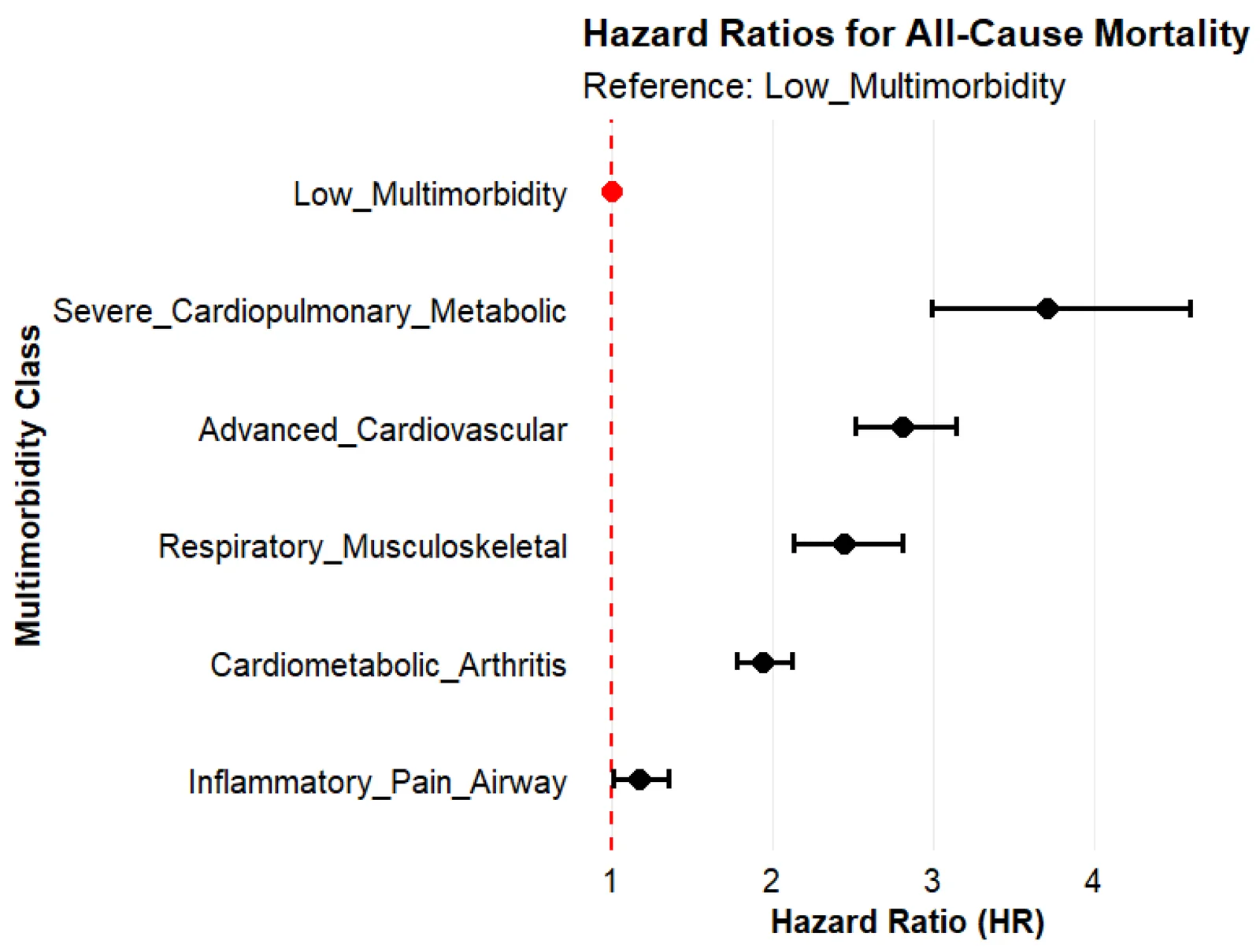
Hazard Ratios for All-cause Mortality for the Fully Adjusted Model.

Further adjustment for behavioral and clinical factors, including smoking, alcohol use, physical activity, sleep duration, and BMI (Model 3), resulted in additional attenuation as shown in Figure 3, although the overall pattern persisted. Class 4 continued to exhibit the highest mortality risk (HR 3.71, 95% CI 2.99–4.60), followed by Class 3 (HR 2.81, 95% CI 2.52–3.14) and Class 1 (HR 2.45, 95% CI 2.13–2.81). Class 2 remained associated with approximately twofold increased risk (HR 1.94, 95% CI 1.78–2.12), whereas Class 5 showed a modest but statistically significant association (HR 1.17, 95% CI 1.01–1.35).

### 3.3. Kaplan–Meier Survival Curves by Latent Class

**Figure 4:**
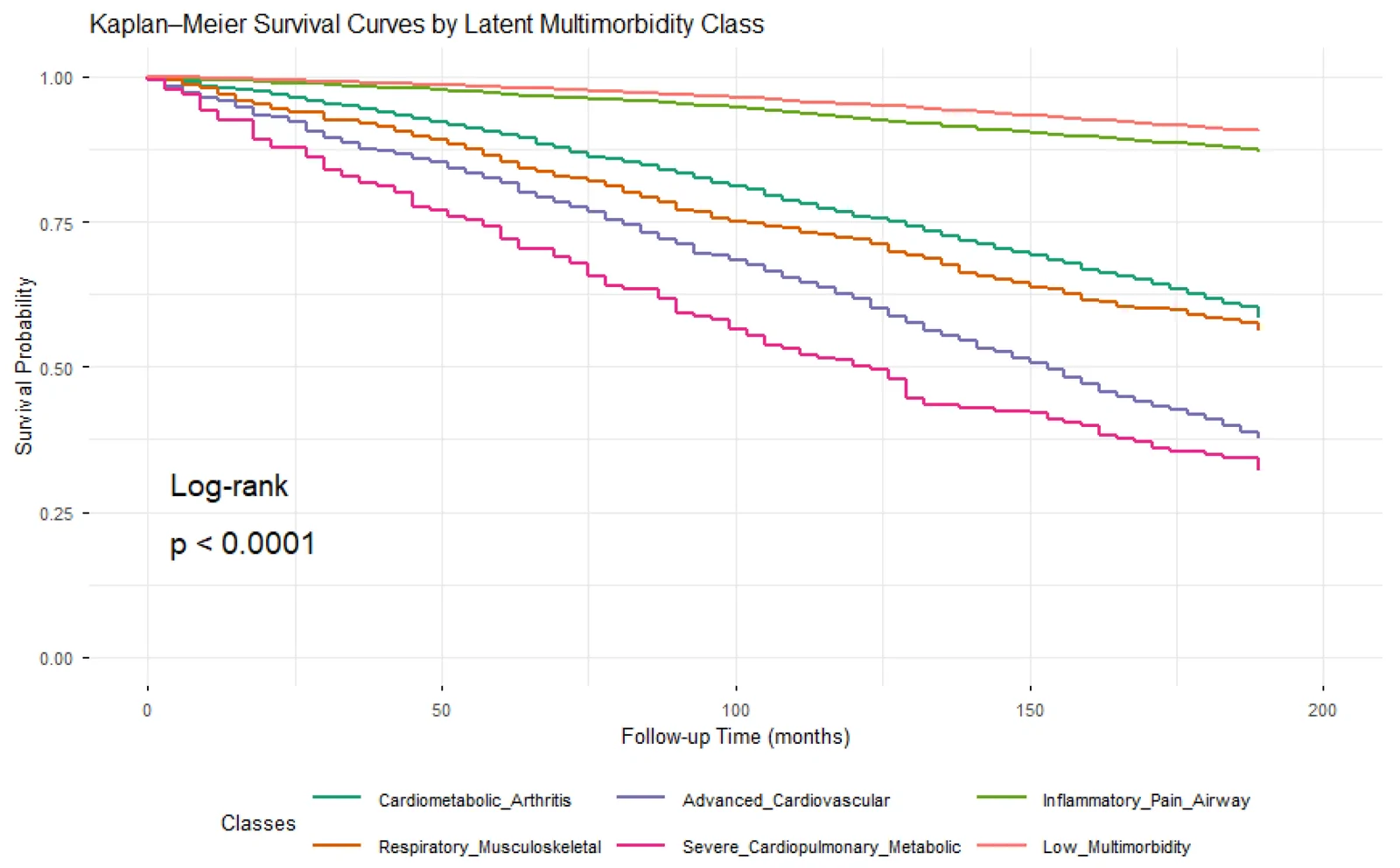
Kaplan–Meier curves by latent class.

Kaplan–Meier survival curves by latent class are presented in Figure 4. Overall, the Low Multimorbidity group maintained the highest survival probability throughout follow-up. In contrast, Classes 3 and 4 exhibited the most pronounced declines in survival, while Classes 1 and 2 demonstrated intermediate survival patterns. The Inflammatory Pain–Airway class showed only a slight separation from the reference group. The log-rank test confirmed significant differences in survival across classes (*p* < 0.0001).

### 3.4. Subgroup Analyses—Age

Age-stratified models revealed that the relative impact of multimorbidity patterns on mortality was strongest in younger adults and attenuated with increasing age (Table 4). Among participants aged 18–39 years, all multimorbidity classes were associated with higher mortality compared with Low Multimorbidity. The Severe Cardiopulmonary–Metabolic class showed the largest relative risk (HR 8.99, 95% CI 1.26–64.15), followed by Advanced Cardiovascular (HR 4.81, 95% CI 1.79–12.94) and Cardiometabolic–Arthritis (HR 3.37, 95% CI 2.19–5.19). Even the Inflammatory Pain–Airway phenotype was associated with 56% higher mortality (HR 1.56, 95% CI 1.08–2.27) in this youngest group.

Sex-specific analyses showed broadly similar associations between multimorbidity classes and mortality in men and women (Table 5). In both sexes, Advanced Cardiovascular and Severe Cardiopulmonary–Metabolic carried the greatest mortality burden. Among men, hazard ratios ranged from 1.62 (95% CI 1.33–1.98) for Inflammatory Pain–Airway to 11.7 (95% CI 8.85–15.5) for Severe Cardiopulmonary–Metabolic, with Cardiometabolic–Arthritis (HR 5.05, 95% CI 4.58–5.57), Respiratory–Musculoskeletal (HR 7.01, 95% CI 5.84–8.42), and Advanced Cardiovascular (HR 8.78, 95% CI 7.82–9.84) showing very strong associations.

### 3.5. Classification Performance Metrics

Classification performance metrics for the six-class solution are shown in Table 6. Average posterior probabilities ranged from 0.637 to 0.869, indicating generally acceptable to strong classification. The highest classification accuracy was observed for Class 5 and Class 6, whereas Class 4 demonstrated lower separation, suggesting potential overlap with other high-burden classes.

## 4. Discussion

### 4.1. Main Findings

Using latent class analysis in a nationally representative sample of U.S. adults, six distinct multimorbidity classes were identified, each characterized by unique patterns of chronic disease. The largest group, the Low Multimorbidity class (68.9%), showed consistently low prevalence across conditions and served as the reference group. In contrast, five higher-burden classes were identified: Cardiometabolic–Arthritis, Respiratory–Musculoskeletal, Advanced Cardiovascular, Severe Cardiopulmonary–Metabolic, and Inflammatory Pain–Airway, each reflecting distinct clustering of cardiometabolic, cardiovascular, respiratory, and inflammatory conditions.

Membership in these multimorbidity classes was associated with substantially elevated mortality risk compared with the Low Multimorbidity group. In the fully adjusted model, the highest risk was observed in the Severe Cardiopulmonary–Metabolic class (HR 3.71), followed by Advanced Cardiovascular (HR 2.81), Cardiometabolic–Arthritis (HR 2.45), and Respiratory–Musculoskeletal (HR 1.94). The Inflammatory Pain–Airway class also demonstrated a modest but statistically significant increase in mortality risk.

Subgroup analyses suggested that the relationship between multimorbidity patterns and mortality varied across populations. The relative impact appeared strongest among younger adults, particularly those aged 18–39 years, implying that the onset of multimorbidity earlier in life may be associated with a greater prognostic burden. Stratification by sex further indicated that increased mortality risk was evident in both men and women; however, the magnitude of this risk was more pronounced among women in the Advanced Cardiovascular and Severe Cardiopulmonary–Metabolic classes.

Overall, these findings highlight that multimorbidity is not only heterogeneous in its structure but also differs in its impact on survival across demographic subgroups.

### 4.2. What Is Already Known

A large body of literature demonstrates that multimorbidity is not randomly distributed but instead forms reproducible clusters of co-occurring conditions across populations [1,2,3,4]. Various analytical approaches, including factor analysis, cluster analysis, and latent class analysis, have consistently identified similar multimorbidity patterns despite methodological differences.

Across settings, studies using factor analysis, cluster analysis, and latent class analysis have identified recurring multimorbidity patterns, including cardiometabolic, mental health, and musculoskeletal profiles [13,31]. These patterns have been observed in diverse populations, including studies conducted in Spain, Korea, and Chile, as well as cross-national analyses, suggesting a broadly consistent structure of multimorbidity globally [22,24,30,31].

Within these clusters, hypertension has consistently emerged as a central condition linking multiple disease patterns. It frequently co-occurs with diabetes, cardiovascular disease, and stroke, reflecting shared vascular and metabolic pathways, and has been identified as one of the most prevalent conditions across multimorbidity classes in several populations, including Brazil and Germany [23,28].

Cardiometabolic and cardiopulmonary clusters have been repeatedly associated with the highest mortality risk, even after adjusting for demographic and behavioral factors [7,14,16,17]. Respiratory conditions often cluster with musculoskeletal disease and functional limitations, contributing to increased vulnerability and adverse outcomes [30]. In addition, inflammatory and pain-related conditions, such as migraine and arthritis, have been linked to systemic inflammation and long-term health risks [29].

Recent work in the United States has expanded this framework. For example, a study by Mukherjee et al. identified five multimorbidity clusters using latent class analysis combined with machine learning, with notable contributions from cardiometabolic and mental health conditions [34]. Similarly, other studies have highlighted the role of mental health disorders as key components of complex multimorbidity, particularly in relation to cardiovascular and metabolic disease pathways.

Furthermore, early-onset multimorbidity has been associated with more severe disease trajectories and higher relative mortality risk, while sex differences in cardiovascular disease presentation and outcomes may contribute to variation in prognosis across men and women [15,17,21,31,32,33].

### 4.3. What This Study Adds

This study builds on existing evidence by providing a more detailed and nuanced characterization of multimorbidity patterns in a nationally representative U.S. population. While previous studies have typically identified two to three classes, and more recent U.S.-based work has identified five clusters incorporating cardiometabolic and mental health conditions [34], our analysis identified six distinct classes, suggesting greater heterogeneity and structural complexity in multimorbidity patterns.

Importantly, our findings demonstrate that cardiometabolic disease is not a single, homogeneous construct but instead comprises distinct high-risk phenotypes. The identification of both Advanced Cardiovascular and Severe Cardiopulmonary–Metabolic classes highlights differences in disease burden and mortality risk that may not be captured when cardiometabolic conditions are grouped together. In addition, the identification of an Inflammatory Pain–Airway class highlights a multimorbidity phenotype that is less consistently reported in prior studies, suggesting potential roles of inflammatory, neurovascular, and environmental mechanisms that may be particularly relevant in the U.S. context.

The inflammatory pattern identified in this study may reflect shared biological pathways underlying multiple chronic conditions rather than isolated disease processes. Chronic low-grade systemic inflammation has been implicated in the development and progression of arthritis, chronic respiratory disease, cardiovascular disease, diabetes, and chronic pain syndromes through persistent immune activation, endothelial dysfunction, and metabolic dysregulation. Environmental exposures, obesity, smoking, physical inactivity, psychosocial stress, and socioeconomic disadvantage may further amplify these inflammatory pathways, contributing to the emergence of multimorbidity clusters. Although the NHIS does not include inflammatory biomarkers, the observed Inflammatory Pain–Airway phenotype is biologically plausible and supports growing evidence that inflammation may represent a common mechanistic link among diverse combinations of chronic diseases.

These findings can be understood considering the underlying health profile of the U.S. population. The United States is characterized by a high prevalence of cardiometabolic risk factors, including hypertension, obesity, and diabetes, as well as substantial burden of respiratory conditions and chronic pain. In addition, mental health conditions and lifestyle factors, such as physical inactivity and smoking, frequently co-occur with these diseases and contribute to more complex multimorbidity patterns, as also highlighted in prior U.S.-based analyses [34]. The coexistence of these factors likely contributes to the emergence of more differentiated and overlapping disease clusters, as observed in the present study.

The subgroup findings further extend the literature by demonstrating that the impact of multimorbidity varies across age and sex groups. The elevated relative risks observed among younger adults underscore the importance of early disease clustering, while the higher risks observed among women in severe classes suggest potential differences in disease progression and healthcare access.

Together, these findings emphasize that multimorbidity should be conceptualized as a heterogeneous, clinically meaningful construct, with important implications for risk stratification and targeted interventions. From a clinical perspective, these findings support moving beyond simple disease counts toward pattern-based risk assessment, enabling clinicians to identify patients with particularly high-risk multimorbidity profiles who may benefit from earlier intervention, closer monitoring, and multidisciplinary care. At the health system level, these multimorbidity patterns may help inform population health management and the development of targeted care pathways for high-risk groups. The Advanced Cardiovascular and Severe Cardiopulmonary-Metabolic classes identified in this study represent patients with particularly complex healthcare needs. Recognizing these multimorbidity patterns may help clinicians and health systems identify individuals who are likely to benefit from coordinated, multidisciplinary care across primary care, cardiology, pulmonology, pharmacy, nutrition, and behavioral health, potentially supported by digital health and remote monitoring technologies. Earlier identification of these high-risk patients may create opportunities for improved continuity of care, enhanced treatment adherence, and more proactive management of patients at elevated risk of adverse outcomes. Our findings also draw attention to the potential value of incorporating multimorbidity patterns into electronic health record (EHR) systems and population health management strategies. Moving beyond simple disease counts, pattern-based assessments may provide a more clinically meaningful way to identify patients with complex healthcare needs who are likely to benefit from enhanced monitoring, care coordination, or chronic disease management. Over time, this approach could strengthen risk stratification and help health systems direct preventive and supportive services to those most likely to benefit.

### 4.4. Possible Explanations for the Observed Patterns

Several biological and social mechanisms may explain the elevated mortality risks observed in the high-morbidity classes.

First, cardiometabolic and cardiopulmonary conditions share common underlying pathways, including chronic inflammation, endothelial dysfunction, and autonomic dysregulation, which may act synergistically to increase mortality risk when these conditions co-occur [35,36]. Second, the coexistence of respiratory and musculoskeletal conditions, as observed in the Respiratory–Musculoskeletal class, may contribute to mortality by reducing mobility, impairing physical function, and increasing susceptibility to infections [18]. Third, the Inflammatory Pain–Airway pattern may reflect dysregulated immune and neurovascular processes. Chronic pain, migraine, and airway inflammation are associated with systemic inflammatory responses and increased physiological stress, which may contribute to long-term adverse outcomes [34].

The particularly high relative risks observed among younger adults likely reflect the severity and rarity of early-onset multimorbidity, which is often linked to cumulative biological stress, delayed diagnosis, and adverse life-course exposures [17,31,32,35]. In older populations, the relative impact of multimorbidity may appear attenuated due to higher baseline mortality risk and competing causes of death [34]. Finally, sex differences in mortality may be influenced by both biological and social factors, including differences in disease presentation, hormonal influences, and access to timely healthcare, particularly for cardiovascular conditions [17,21,33].

### 4.5. Strengths and Limitations

This study benefits from the use of nationally representative U.S. data, enabling generalizability across diverse demographic and socioeconomic populations. Latent class analysis identified clinically coherent disease clusters, and mortality associations were assessed using robust survival models with subgroup analyses.

Several limitations should be considered when interpreting these findings. First, chronic conditions were based on self-reported data, which may be subject to recall bias and misclassification, potentially leading to underestimation or overestimation of disease prevalence. Second, the set of chronic conditions included in the analysis may influence the number and composition of the latent classes identified; different disease selections could yield alternative class structures. Third, multimorbidity was assessed only at baseline, and changes in disease status over time were not captured, limiting our ability to examine transitions between classes and the dynamic progression of multimorbidity.

Additionally, information on age at disease onset was not available, which is important given that early-onset multimorbidity may have different trajectories and mortality implications compared to later-life onset. Residual confounding is also possible, as unmeasured factors such as healthcare access, treatment adherence, environmental exposures, and psychosocial stressors may influence both class membership and mortality risk. Finally, as with all observational studies, causal inferences cannot be established; the observed associations should be interpreted accordingly. Future studies should evaluate whether these multimorbidity classes can be incorporated into clinical decision support tools embedded within electronic health records for automated risk stratification and validated prospectively in healthcare settings.

## 5. Conclusions

In a nationally representative sample of U.S. adults, multimorbidity was classified into six distinct, clinically meaningful classes, with substantial heterogeneity in disease patterns and mortality risk. Classes characterized by cardiometabolic and cardiopulmonary conditions exhibited the highest mortality, while additional patterns, including inflammatory pain–airway profiles, also demonstrated elevated risk. Hypertension emerged as a central condition across high-burden classes, underscoring its role in the development and progression of multimorbidity.

These findings highlight that multimorbidity should not be viewed as a simple accumulation of diseases but as a set of interconnected condition clusters with distinct prognostic implications. Recognizing these patterns can improve risk stratification, guide targeted prevention strategies, and support more integrated, patient-centered approaches to care. In the United States, where multimorbidity is both prevalent and complex, such approaches are essential for reducing disease burden and improving population health outcomes.

Beyond their prognostic significance, these findings have important implications for clinical practice and population health. Our findings suggest that clinically meaningful patterns of multimorbidity may offer greater value than simple disease counts for identifying patients at increased risk of death. Future research should determine whether using these patterns in routine clinical practice and population health management can improve care delivery and patient outcomes. These findings also have important implications for healthcare policy by supporting the prioritization of preventive services and coordinated chronic disease management for individuals with high-risk multimorbidity profiles. Integrating pattern-based multimorbidity assessment into routine clinical practice and population health planning may improve resource allocation and ultimately reduce premature mortality.

## Figures and Tables

**Table 1:** Prevalence (%) of chronic conditions overall and across latent multimorbidity classes.

Chronic Disease	General Population	Class 1	Class 2	Class 3	Class 4	Class 5	Class 6
		Cardiometabolic Arthritis	Respiratory Musculoskeletal	Advanced Cardiovascular	Severe Cardiopulmonary Metabolic	Inflammatory Pain Airway	Healthy Class
Diabetes	7.7	29.3	21.0	30.2	34.0	0.0	1.6
Stomach/Intestinal Ulcer	7.3	12.2	30.4	15.9	47.3	30.2	1.6
Coronary Heart Disease	4.5	0.0	1.7	86.4	79.8	0.1	0.2
Angina	2.6	2.7	6.6	31.2	63.3	1.2	0.1
Myocardial Infarction	3.4	2.7	4.9	53.3	62.8	0.0	0.2
Hypertension	26.9	83.1	71.9	71.5	87.8	6.4	11.6
Stroke	2.7	8.6	13.9	16.6	41.0	0.0	0.0
Heart Failure	7.6	16.6	25.7	42.4	66.0	12.7	1.5
Asthma	10.3	4.9	75.7	6.2	63.3	39.0	5.1
Chronic Bronchitis	4.6	2.8	60.8	3.9	59.6	22.1	0.0
Emphysema	1.8	1.0	28.4	5.1	44.7	2.6	0.1
Arthritis	22.5	63.0	71.1	50.3	87.8	43.6	6.6
Hepatitis	3.2	6.3	8.8	4.2	10.6	4.8	2.0
Migraine	16.0	12.3	41.5	11.1	45.7	62.6	10.4
Cancer (any)	7.2	23.1	18.2	18.9	16.5	12.2	1.8

Note: Values in the “General population” column (Table 1) represent observed prevalence percentages. Values for latent classes represent conditional item-response probabilities (%) derived from latent class analysis (LCA).

**Table 2:** Baseline sociodemographic, behavioral, and clinical characteristics across latent multimorbidity classes.

Variable	Class 1	Class 2	Class 3	Class 4	Class 5	Class 6
Sample size (%)	4323 (15.1)	873 (3.1)	1250 (4.4)	188 (0.7)	2269 (7.9)	19,695 (68.9)
Age, mean (SD)	61.26 (14.02)	57.38 (15.16)	66.08 (12.22)	64.03 (11.99)	43.64 (15.26)	41.25 (15.08)
**Sex, % (95% CI)**						
Male	41.1 (39.6, 42.5)	30.4 (27.3, 33.4)	58.2 (55.4, 60.9)	40.4 (33.4, 47.4)	31.1 (29.2, 33.0)	48.0 (47.3, 48.7)
Female	58.9 (57.5, 60.4)	69.6 (66.6, 72.7)	41.8 (39.1, 44.6)	59.6 (52.6, 66.6)	68.9 (67.0, 70.8)	52.0 (51.3, 52.7)
**Race/Ethnicity, % (95% CI)**						
Black/African-American	16.1 (15.0, 17.1)	15.1 (12.7, 17.5)	9.9 (8.3, 11.6)	13.3 (8.4, 18.2)	12.2 (10.8, 13.5)	14.1 (13.6, 14.6)
Other	3.8 (3.2, 4.4)	4.4 (3.0, 5.7)	3.8 (2.8, 4.9)	2.7 (0.4, 5.0)	4.5 (3.7, 5.4)	5.6 (5.2, 5.9)
White	80.1 (78.9, 81.3)	80.5 (77.9, 83.2)	86.2 (84.3, 88.1)	84.0 (78.8, 89.3)	83.3 (81.8, 84.8)	80.4 (79.8, 80.9)
**Marital Status, % (95% CI)**						
Married	47.0 (45.5, 48.5)	39.7 (36.5, 43.0)	53.7 (50.9, 56.4)	35.1 (28.3, 41.9)	45.4 (43.3, 47.4)	48.8 (48.1, 49.5)
Separated/Divorced/Widowed	42.5 (41.1, 44.0)	48.5 (45.1, 51.8)	39.6 (36.9, 42.3)	55.3 (48.2, 62.4)	28.7 (26.9, 30.6)	21.5 (20.9, 22.1)
Single/Never-Married	10.4 (9.5, 11.3)	11.8 (9.7, 13.9)	6.7 (5.3, 8.1)	9.6 (5.4, 13.8)	25.9 (24.1, 27.7)	29.7 (29.0, 30.3)
**Smoking Status, % (95% CI)**						
Smoking, Unclear	13.1 (12.1, 14.1)	27.5 (24.5, 30.5)	14.0 (12.1, 15.9)	27.1 (20.8, 33.5)	26.4 (24.5, 28.2)	16.8 (16.3, 17.3)
Current Smoker	3.0 (2.5, 3.5)	4.0 (2.7, 5.3)	2.4 (1.6, 3.2)	5.3 (2.1, 8.5)	5.1 (4.2, 6.0)	4.8 (4.5, 5.1)
Former Smoker	31.8 (30.4, 33.2)	33.9 (30.8, 37.0)	45.1 (42.4, 47.9)	41.5 (34.4, 48.5)	21.6 (19.9, 23.2)	16.4 (15.9, 16.9)
Never Smoker	52.0 (50.6, 53.5)	34.6 (31.4, 37.7)	38.5 (35.8, 41.2)	26.1 (19.8, 32.3)	47.0 (45.0, 49.1)	62.0 (61.4, 62.7)
**Alcohol Intake, % (95% CI)**						
Current	27.0 (25.7, 28.3)	24.5 (21.7, 27.5)	27.5 (25.1, 30.1)	26.6 (20.4, 33.5)	19.8 (18.2, 21.5)	26.3 (25.6, 27.1)
Former	23.8 (22.5, 25.1)	28.1 (25.1, 31.2)	30.2 (27.6, 32.8)	43.1 (35.9, 50.5)	16.8 (15.3, 18.4)	10.9 (10.4, 11.4)
Never	49.2 (47.7, 50.7)	47.4 (44.1, 50.8)	42.3 (39.6, 45.1)	30.3 (23.8, 37.4)	63.4 (61.4, 65.4)	62.8 (62.0, 63.6)
**Physical Activity, % (95% CI)**						
Vigorous activities	13.2 (12.2, 14.2)	15.8 (13.4, 18.2)	14.8 (12.8, 16.8)	19.7 (14.0, 25.4)	12.2 (10.8, 13.5)	10.0 (9.6, 10.5)
Light/Mod	40.6 (39.1, 42.1)	32.3 (29.2, 35.4)	36.9 (34.2, 39.6)	24.5 (18.3, 30.6)	44.9 (42.9, 47.0)	43.8 (43.1, 44.5)
Never/Unable	46.2 (44.7, 47.7)	51.9 (48.6, 55.2)	48.3 (45.5, 51.1)	55.9 (48.8, 62.9)	42.9 (40.9, 45.0)	46.2 (45.5, 46.9)
**BMI Category, % (95% CI)**						
Normal	25.4 (24.1, 26.7)	27.3 (24.3, 30.2)	27.9 (25.4, 30.4)	24.5 (18.3, 30.6)	41.7 (39.7, 43.7)	44.0 (43.3, 44.7)
Pre-Obese	36.1 (34.7, 37.6)	29.0 (26.0, 32.0)	38.6 (35.9, 41.3)	38.3 (31.3, 45.2)	31.4 (29.5, 33.3)	35.7 (35.0, 36.4)
Obese	38.5 (37.0, 39.9)	43.8 (40.5, 47.0)	33.5 (30.9, 36.1)	37.2 (30.3, 44.1)	26.9 (25.1, 28.7)	20.3 (19.7, 20.8)
**Sleep Duration Category, % (95% CI)**						
Sleep ≤ 4 h	3.4 (2.9, 4.0)	10.1 (8.1, 12.1)	4.8 (3.6, 6.0)	13.3 (8.4, 18.2)	4.9 (4.0, 5.8)	1.6 (1.4, 1.7)
Sleep 5, 6 h	28.5 (27.1, 29.8)	31.2 (28.1, 34.2)	27.5 (25.0, 30.0)	29.8 (23.2, 36.3)	33.8 (31.8, 35.7)	25.8 (25.2, 26.4)
Sleep 7, 8 h	57.4 (55.9, 58.9)	40.0 (36.7, 43.2)	53.2 (50.4, 56.0)	38.8 (31.9, 45.8)	52.3 (50.2, 54.3)	65.7 (65.1, 66.4)
Sleep 9 h	5.5 (4.8, 6.1)	8.2 (6.4, 10.1)	6.8 (5.4, 8.2)	7.4 (3.7, 11.2)	4.9 (4.0, 5.8)	3.9 (3.7, 4.2)
Sleep ≥ 10 h	5.2 (4.6, 5.9)	10.5 (8.5, 12.6)	7.7 (6.2, 9.2)	10.6 (6.2, 15.0)	4.1 (3.3, 5.0)	2.9 (2.7, 3.2)

Abbreviations: SD, standard deviation; BMI, body mass index; CI, confidence interval.

**Table 3:** Association between latent multimorbidity classes created and all-cause mortality.

Class (vs. Class 6)	Model 1 (Unadjusted)		Model 2 (Adjusted for Sociodemographic Factors)		Model 3 (Fully Adjusted for Behavioral and Lifestyle Covariates)	
	HR (95% CI)	*p*-Value	HR (95% CI)	*p*-Value	HR (95% CI)	*p*-Value
Class 6 (reference)	1.00 (ref.)	–	1.00 (ref.)	–	1.00 (ref.)	–
Class 1	5.95 (5.31–6.68)	<0.001	2.70 (2.41–3.04)	<0.001	2.45 (2.13–2.81)	<0.001
Class 2	5.26 (4.91–5.62)	<0.001	1.97 (1.83–2.11)	<0.001	1.94 (1.78–2.12)	<0.001
Class 3	9.71 (8.90–10.6)	<0.001	2.99 (2.72–3.27)	<0.001	2.81 (2.52–3.14)	<0.001
Class 4	12.3 (10.2–14.7)	<0.001	4.10 (3.41–4.94)	<0.001	3.71 (2.99–4.60)	<0.001
Class 5	1.39 (1.22–1.58)	<0.001	1.29 (1.13–1.46)	<0.001	1.17 (1.01–1.35)	0.0386

**Table 4:** Association between latent multimorbidity classes and all-cause mortality stratified by age group.

Multimorbidity Class	Age 18–39		Age 40–59		Age 60+	
	HR (95% CI)	*p*-Value	HR (95% CI)	*p*-Value	HR (95% CI)	*p*-Value
Cardiometabolic–Arthritis	3.37 (2.19–5.19)	<0.001	2.48 (2.15–2.86)	<0.001	1.72 (1.58–1.87)	<0.001
Respiratory–Musculoskeletal	2.80 (1.32–5.94)	0.007	4.32 (3.49–5.34)	<0.001	2.15 (1.87–2.48)	<0.001
Advanced Cardiovascular	4.81 (1.79–12.94)	0.019	4.17 (3.34–5.20)	<0.001	2.72 (2.46–3.01)	<0.001
Severe CPM	8.99 (1.26–64.15)	0.028	9.90 (7.07–13.87)	<0.001	3.19 (2.56–3.98)	<0.001
Inflammatory Pain–Airway	1.56 (1.08–2.27)	<0.001	1.16 (0.92–1.45)	0.075	1.23 (1.03–1.46)	<0.001

Abbreviations: CPM, Cardiopulmonary-Metabolic; HR, hazard ratio; CI, confidence interval. Note: Models were adjusted for all covariates except age.

**Table 5:** Sex Association between latent multimorbidity classes and all-cause mortality stratified by sex.

Multimorbidity Class	Male		Female	
	HR (95% CI)	*p*-Value	HR (95% CI)	*p*-Value
Cardiometabolic–Arthritis	5.05 (4.58–5.57)	<0.001	5.64 (5.12–6.20)	<0.001
Respiratory–Musculoskeletal	7.01 (5.84–8.42)	<0.001	5.93 (5.11–6.89)	<0.001
Advanced Cardiovascular	8.78 (7.82–9.84)	<0.001	10.70 (9.33–12.2)	<0.001
Severe CPM	11.70 (8.85–15.5)	<0.001	13.30 (10.4–16.9)	<0.001
Inflammatory Pain–Airway	1.62 (1.33–1.98)	<0.001	1.36 (1.15–1.61)	<0.001

Abbreviations: CPM, Cardiopulmonary-Metabolic; HR, hazard ratio; CI, confidence interval.

**Table 6:** Classification performance metrics for the six-class latent class analysis model.

Class	AvePP	OCC	Interpretation
Class 1	0.723	79.671	Acceptable classification
Class 2	0.767	18.954	Good class separation
Class 3	0.784	17.214	Good class separation
Class 4	0.637	1.554	Weak classification
Class 5	0.869	139.7	Excellent class separation
Class 6	0.805	515.576	Strong classification accuracy

Abbreviations: AvePP, average posterior probability; OCC, odds of correct classification.

## Data Availability

The data used in this study are publicly available from the National Health Interview Survey (NHIS) and the Linked Mortality Files provided by the US National Center for Health Statistics (NCHS). Data can be accessed through the NCHS Research Data Center or downloaded from the NHIS website: https://www.cdc.gov/nchs/nhis/ access on 25 February 2026.

## Supplementary Materials

The following supporting information can be downloaded at: https://www.mdpi.com/article/10.3390/diseases14080270/s1, Table S1: Comparison of baseline characteristics between the full eligible sample and the final analytic sample.

## Abbreviations

The following abbreviations are used in this manuscript:

NCDs	Non-Communicable Diseases
LCA	Latent Class Analysis
NCHS	National Center for Health Statistics
NHIS	National Health Interview Survey
NDI	National Death Index
