# Supplementary material for: Multimorbidity Patterns and Mortality Risk in a National Sample of US Adults Identified Using Latent Class Analysis
Source: Diseases. 2026 Jul 24;14(8):270. doi: 10.3390/diseases14080270 (PMC13511899; doi:10.3390/diseases14080270)
Supplement: Supplementary file 1 [file diseases-14-00270-s001.zip › diseases-4380610-supplementary.pdf]

Table S1. Comparison of baseline characteristics between the full eligible sample and the final analytic sample

Variable	Full eligible sample (N = 60,290)	Final analytic sample (N = 28,598)
Age, mean (SD)	55.61(53.6, 56.7)	52.58(51.2, 53.9)
Sex, % (95% CI)		
Male	48.1 (47.8, 48.4)	46.4 (45.8, 47.1)
Female	51.9 (51.6, 52.2)	53.6 (52.9, 54.2)
Race/Ethnicity, % (95% CI)		
White	83.2 (82.6, 83.9)	83.0 (82.3, 83.7)
Black/African American	11.4 (10.8, 12.0)	11.9 (11.3, 12.6)
Other	5.4 (5.0, 5.7)	5.0 (4.7, 5.4)
Education, % (95% CI)		
Less than high school	16.6 (16.0, 17.1)	15.6 (15.0, 16.2)
High school	30.0 (29.4, 30.6)	28.2 (27.5, 28.9)
More than high school	53.4 (52.7, 54.2)	56.2 (55.2, 57.1)
Marital status, % (95% CI)		
Married	58.4 (57.7, 59.0)	48.5 (47.7, 49.3)
Separated/Divorced/Widowed	18.8 (18.3, 19.2)	26.5 (25.8, 27.2)
Single/Never married	22.9 (22.3, 23.5)	25.0 (24.1, 25.9)
Smoking status, % (95% CI)		
Current smoker	5.1 (4.8, 5.4)	5.3 (4.9, 5.6)
Former smoker	27.1 (26.4, 27.7)	27.1 (26.4, 27.8)
Never smoker	67.8 (67.1, 68.5)	67.6 (66.9, 68.4)
Alcohol intake, % (95% CI)		
Current	23.2 (22.5, 23.9)	22.2 (21.5, 23.0)
Former	15.2 (14.7, 15.7)	15.0 (14.5, 15.5)
Never	61.6 (60.7, 62.4)	62.8 (62.0, 63.6)
BMI category, % (95% CI)		
Normal/Underweight	71.9 (71.5, 72.3)	40.4 (39.7, 41.1)
Pre-obese	16.6 (16.3, 16.9)	35.2 (34.6, 35.7)
Obese	11.4 (11.1, 11.7)	24.4 (23.8, 25.0)
Sleep duration, % (95% CI)		
⩽4 h	2.5 (2.3, 2.7)	2.5 (2.3, 2.7)
5–6 h	27.0 (26.4, 27.6)	27.2 (26.6, 27.8)
7–8 h	61.8 (61.1, 62.5)	61.9 (61.2, 62.7)
9 h	4.7 (4.4, 4.9)	4.6 (4.3, 4.9)
⩾10 h	4.0 (3.8, 4.3)	3.8 (3.5, 4.0)

Abbreviations: BMI, body mass index; CI, confidence interval.
